# High-Value Utilization of Coconut Kernel Fiber By-Products: The Insulin-Sensitizing Effect of Novel α-Glucosidase-Inhibiting Peptides Derived from Coconut Kernel Fiber on T2DM Mice

**DOI:** 10.3390/foods15122105

**Published:** 2026-06-11

**Authors:** Dingyan Sun, Xiaoshan Zheng, Mingliang Zhang, Jiemin Pan, Ying Lu

**Affiliations:** 1 College of Food Science and Technology, Shanghai Ocean University, Shanghai 201306, China; 15190973118@163.com (D.S.); zxs961023@163.com (X.Z.); 2 National R&D Branch Center for Freshwater Aquatic Products Processing Technology (Shanghai), Shanghai 201306, China; 3 Department of Endocrinology and Metabolism, Haikou Orthopedic and Diabetes Hospital, Haikou 570311, China; 4 Department of Endocrinology and Metabolism, Shanghai Sixth People’s Hospital, Shanghai 200233, China; lilyriku@sjtu.edu.cn; 5 Marine Biomedical Science and Technology Innovation Platform of Lingang Special Area, Shanghai 201306, China

**Keywords:** coconut kernel fiber, α-glucosidase inhibitory peptide, insulin resistance, Type 2 diabetes mellitus, hypoglycemic foods

## Abstract

Coconut kernel fiber (CKF) is a by-product of coconut oil processing; it is rich in protein and serves as a potential source of bioactive peptides. In this study, from the enzymatic hydrolysis products of CKF (CKFH), a low-molecular-weight CKFH component (LW-CKFH, 1–3 kDa), exhibiting 74.49% α-glucosidase inhibition and restoring glucose metabolism in IR-HepG2 cells to 71.37% of normal levels. In a type 2 diabetes (T2DM) mouse model, LW-CKFH alleviated insulin resistance and enhanced insulin sensitivity by repairing liver damage, thereby improving glucose and lipid metabolism and reducing inflammation; its effects on improving insulin resistance and sensitivity reached 75.43% and 75.47% of the efficacy of metformin, respectively. Molecular docking analysis identified FDLPAR, LPFPRPAGPR, and ANVFNPR as key active peptides responsible for inhibiting α-glucosidase activity. Furthermore, LW-CKFH exhibited good gastrointestinal digestibility and processing stability, while significantly reducing the glucose release rate from bread (>50%), indicating its suitability for the development of hypoglycemic or low-GI functional foods. LW-CKFH was particularly suitable as a functional ingredient for fruits, vegetables, grains, and dairy products to develop low-GI or hypoglycemic foods. This study provides new insights into the high-value utilization of the coconut processing by-product CKF.

## 1. Introduction

Type 2 diabetes (T2DM) is a serious disorder of glucose metabolism caused by insufficient insulin secretion and insulin resistance. To date, the number of people with T2DM worldwide has risen to approximately 600 million, and incidence and prevalence continue to rise in most countries, particularly in low- and middle-income countries [1]. Persistent hyperglycemia can lead to a variety of complications, thereby endangering the lives of patients [2]. Common chemically synthesized drugs used in clinical practice (such as acarbose and sitagliptin) can cause side effects such as gastrointestinal adverse reactions—including bloating and diarrhea—when taken long-term, and they place a significant financial burden on patients. Therefore, there is an urgent need to develop highly effective and safe hypoglycemic agents derived from natural sources to explore new avenues for dietary intervention and adjunctive therapy in diabetes.

Due to their high safety profile and good biocompatibility, natural bioactive peptides have become an important adjunct to conventional medications. Among these, food-derived bioactive peptides have demonstrated significant therapeutic potential in this field. For example, Santo-Hernández et al. found that whey protein peptides could promote insulin secretion by inhibiting DPP-IV activity, thereby improving glucose metabolism in patients with type 2 diabetes mellitus [3]; Xing et al. discovered that the active peptide VAMP derived from hemp seeds inhibited DPP-IV activity through competitive binding and specifically promoted the proliferation of *Akkermansia* muciniphila, achieving a dual mechanism to improve glucose metabolism disorders [4]. Currently, DPP-IV inhibitory peptides have been systematically studied at the molecular, cellular, and animal levels regarding their efficacy evaluation, structure–activity relationships, and mechanisms of hypoglycemic action and the regulation of the gut microbiota [5]. The α-glucosidase exerts its physiological effect by specifically acting on the carbohydrate digestion in the intestine, That is, it can delay the enzymatic hydrolysis of complex carbohydrates into glucose by inhibiting the activity of α-glucosidase. thereby reducing the production of glucose. Therefore, for individuals with a high-carbohydrate diet, the α-glucosidase inhibitory peptides can not only reduce the post-meal blood glucose fluctuations, but can also alleviate the insulin secretion stress on pancreatic β cells, which is beneficial for post-meal blood glucose regulation in T2DM patients. Thus, α-glucosidase inhibitors are an important clinical therapeutic tool for T2DM.

It was reported by Fadimu et al. [6] that the active peptides from lupin could effectively slow down the rate of glucose production by inhibiting the activity of α-glucosidase, thereby alleviating the abnormal glucose metabolism caused by T2DM. Li et al. extracted the active peptides SSPP from the seed meal of sea buckthorn, and found that it had high inhibitory activity of α-glucosidase with the half-inhibitory concentration (IC_50_) as low as 0.18 mg/mL, which was significantly superior to the clinical drug acarbose [7]. Zhang et al. obtained α-glucosidase inhibitory peptides from the enzymatic hydrolysis products of hemp seed. They discovered that the binding of this peptide segment to the active site of α-glucosidase not only prevented the catalytic reaction, but also blocked the substrate from entering the active site of the enzyme [8]. Wang et al. identified two new inhibitory peptides, FNSL and DVTPL from the hydrolysate of mung bean protein, with IC_50_ values of 0.24 mmol/L and 0.27 mmol/L respectively, which were close to acarbose. Molecular dynamics simulation further revealed that the binding free energy of the peptide segment was −12.773 kcal/mol, and it interacted with α-glucosidase mainly through hydrogen bonds, salt bridges, and van der Waals forces [9]. Hau et al. identified seven novel α-glucosidase inhibitory peptides from hydrolysates of oil palm leaves and confirmed that these peptides form stable complexes with α-glucosidase primarily through hydrogen bonds and salt bridges [10]. Although researchers have isolated and identified peptides capable of inhibiting α-glucosidase activity from various plant sources, there is a lack of systematic research on the hypoglycemic mechanism of α-glucosidase inhibitory peptides and their regulation of blood glucose homeostasis in T2DM.

In recent years, food processing by-products have attracted significant attention due to their low cost and high protein content, making them a current research hotspot for the discovery of naturally occurring bioactive peptides. For example, defatted castor bean meal, the primary by-product of castor oil extraction with high protein content of 64.54%. Bian et al. successfully isolated peptides with good α-glucosidase inhibitory activity from it. Among them, AAGF, QPTM, and QGSM bound to the enzyme active site through hydrogen bonds and exhibited good thermal stability and acid stability, making them suitable as functional food ingredients [11]. Zhou et al. used peanut meal as the raw material, and isolated active peptide fractions ranging from 3 kDa to 500 Da through enzymatic hydrolysis combined with ultrafiltration, which exhibited an α-glucosidase inhibitory activity up to 69.5%. They found that high dose of the active peptides effectively reduced blood glucose and lipid levels, alleviated liver damage and symptoms of T2DM mice [12].

Coconut kernel fiber (CKF) is the dietary fiber obtained from the white endosperm (kernel) of coconut after oil or milk extraction, and offers advantages such as low cost and easy availability. It has been reported that CKF contains approximately 23% protein [13], suggesting it is a potential source of bioactive peptides. Currently, CKF by-products are primarily utilized in low-value-added applications, which hinders its sustainable development. Therefore, this study aims to explore the bioactive peptides of CKF and clarify their efficacy and application potentials. Firstly, enzymatic hydrolysis of CKF (CKFH) was prepared, and the low molecular weight CKFH (LW-CKFH) with high inhibitory activity against α-glucosidase was screened. Next, in vitro cellular models and in vivo animal models were employed to investigate the hypoglycemic effects of these active peptides. In addition, the processing stability including the effect of food matrices, pH, thermal temperature and gastrointestinal digestion on the activity of LW-CKFH were investigated. Furthermore, we also investigated whether LW-CKFH could slow down the generating rate of glucose from the starch in bread, in order to assess the potential application of the active peptide. Based on the identified LW-CKFH structure combining bioinformatics tools, three highly active oligopeptides were screened and synthesized. The interaction between the oligopeptides and α-glucosidase was also studied. This study provides a scientific basis for the high-value utilization of CKF and offers new strategies for the development of functional foods that help control postprandial blood glucose.

## 2. Materials and Methods

### 2.1. Materials

CKF is the dietary fiber obtained from the white endosperm (kernel) of coconut after oil or milk extraction. The CKF sample was provided by Shanghai Children’s Nutrition Center Co., Ltd. (Haikou, China). All six commercial proteases were purchased from Genclone Biotechnology Co., Ltd. (Beijing, China). The EC numbers of the enzymes are as follows: neutral protease (EC 3.4.24.28), alkaline protease (EC 3.4.21.62), flavor protease (EC 3.4.11.1), papain (EC 3.4.22.2), trypsin (EC 3.4.21.4), cellulase (EC 3.2.1.4), and α-glucosidase (EC 3.2.1.20). α-Glucosidase, p-nitrophenyl-β-D-glucoside (pNPG), and acarbose were purchased from Shanghai Yuanye Biotechnology Co., Ltd. (Shanghai, China). Insulin and metformin hydrochloride were obtained from Beijing Solang Biotechnology Co., Ltd. (Beijing, China). Streptozotocin (STZ) was purchased from Beijing Laibost Technology Co., Ltd. (Beijing, China). The human HepG-2 cell line was purchased by the Chinese Academy of Sciences (Shanghai, China). The glucose colorimetric test kit was provided from Wuhan Islate Co., Ltd. (Wuhan, China). Insulin (INS), triglycerides (TG), total cholesterol (TC), high-density lipoprotein cholesterol (HDL-C), low-density lipoprotein cholesterol (LDL-C), and glycated serum protein (GSP) test kits were purchased from Yancheng Junxing Biotechnology Co., Ltd. (Yancheng, China). The three peptides were synthesized by Nanjing Genscript Biotech Co., Ltd. (Nanjing, China).

### 2.2. Preparation of CKFH

The enzymatic hydrolysis of CKF was performed using the method described by Wang et al. [14] with slight modifications. The specific procedure was as follows: After thoroughly mixing CKF powder with 3% (*w*/*v*) distilled water, alkaline protease (pH 10.0, 55 °C), neutral protease (pH 7.0, 45 °C), trypsin (pH 7.0, 37 °C), flavor protease (pH 7.5, 55 °C), papain (pH 7.0, 55 °C), and cellulase (pH 5.0, 50 °C) were each added at a dosage of 8000 U/g, and each was allowed to react for 4 h for single-enzyme hydrolysis. The dual-enzyme combinations were neutral enzyme and alkaline enzyme, neutral enzyme and pancreatic enzyme, and alkaline enzyme and pancreatic enzyme, with each enzyme added at 8000 U/g in a 1:1 ratio. The hydrolysis system was adjusted to the required pH and temperature according to the product manual, and the reaction was carried out for 4 h. For combined ultrasonic treatment with two enzymes, 8000 U/g of neutral protease was first added, and the sample was placed in an ultrasonic device (L-UCS-10L, Beijing Lanjieke Technology Co., Ltd., Beijing, China) at 45 °C for 2 h, followed by enzyme inactivation. Next, 8000 U/g of alkaline protease was added to a pH 10 solution, and the mixture was subjected to ultrasonication at 55 °C for 2 h (ultrasonic frequency 40 kHz, power 300 W). The reaction product was then boiled for 10 min, after which the pH was adjusted to 7.0. It was subsequently centrifuged at 8000 rpm for 10 min. The resulting supernatant was freeze-dried under vacuum, and the obtained powder was used as the CKFH sample for subsequent experiments.

### 2.3. Isolation and Purification of Highly Active CKFH Fractions

CKFH solution was separated by centrifugal filter with molecular weight cut-offs of 10 kDa, 3 kDa, and 1 kDa. After centrifugation at 8000 rpm for 10 min, the isolated fractions were subjected to hypoglycemic activity testing. After identifying the optimal hypoglycemic fraction, it was freeze-dried (SCIENTZ-18 N, Bilang Instrument Manufacturing, Shanghai, China). After ultrafiltration, the fraction exhibiting the highest α-glucosidase inhibitory activity (10 mg/mL) was filtered through a 0.45 µm membrane. This fraction was then separated using a G-25 dextran gel at a flow rate of 0.5 mL/min. Absorbance was measured at 220 nm to generate an elution curve, and the α-glucosidase inhibitory activity of each peak was determined.

### 2.4. Analysis of α-Glucosidase Inhibitory Activity

The method for determining the α-glucosidase inhibitory activity of the sample was adapted from Kim et al. [15] with minor modifications incorporating the procedures described by Lu et al. [16]. In brief, 30 μL aliquot of the sample was mixed with 100 μL of 0.1 U/mL α-glucosidase solution in phosphate-buffered saline (PBS, pH 6.8) and incubated at 37 °C for 10 min. Subsequently, 30 μL of 2.5 mmol/L pNPG solution was added and incubated at 37 °C for 20 min. Next, 100 μL of sodium carbonate solution (0.2 mol/L) was added, and the absorbance at 405 nm of the mixture solution was measured by a microplate reader (SuPerMax 3200, Shanghai Flash Spectrum Biological Technology Co., Ltd, Shanghai, China). The logarithm of the concentrations of acarbose and the isolated purified component (F2) was plotted against the inhibition rate, and a nonlinear fit (four-parameter logistic model, 4PL) was performed.

(1) $$\mathrm{Inhibition}\;\mathrm{rate}\;\mathrm{of}\;\alpha \text{-}\mathrm{glucosidase}\;\mathrm{activity}\;(\%)=(1-\frac{A_{S}-A_{b}}{A_{c}})\times 100$$

where *A_s_* represented the absorbance of the mixed solution containing sample and α-glucosidase. *A_b_* was the absorbance of the control solution where α-glucosidase solution was replaced by PBS. *A_c_* was the absorbance of the control solution where the sample was replaced by PBS.

The control group consisted of an untreated LW-CKFH solution (20 mg/mL, pH 7.0, 25 °C), which received no treatment (i.e., no addition of metal ions, no food matrix, and no gastrointestinal digestion). Its α-glucosidase inhibitory activity was set at 100%. Subsequently, the α-glucosidase inhibitory activity of the treated samples was measured under the same detection conditions and compared with the control group.

(2) $$\mathrm{Retention}\;\mathrm{rate}\;\mathrm{of}\;\alpha \text{-}\mathrm{glucosidase}\;\mathrm{inhibitory}\;\mathrm{activity}\;(\%)=(\frac{I_{2}}{I_{1}})\times 100$$

where *I*_2_ represented the α-glucosidase inhibition rate of the treated sample; *I*_1_ was the α-glucosidase inhibition rate of the control sample (100%).

### 2.5. Structural Characterization of CKF and Its Hydrolysate

The microstructure of CKF, ultrasonicated CKF (UCKF), CKFH, and LW-CKFH were observed using a scanning electron microscope (SEM). Each sample powder was secured to the sample stage using double-sided carbon tape and spread out evenly. After gold sputtering, the samples were placed in a high-resolution field-emission scanning electron microscope (Hitachi SU5000, Hitachi High-Tech Corporation, Tokyo, Japan) and imaged at an acceleration voltage of 5 kV.

### 2.6. IR-HepG2 Cell Model Construction and Its Glucose Consumption Analysis

Cell viability was assessed using the MTT assay [17]. IR-HepG2 cell model was constructed according to the method established in our previous study [18]. HepG2 cells in the logarithmic growth phase were plated in a 96-well plate at a density of 1 × 10^4^ cells per well and incubated for 24 h (37 °C, 5% CO_2_) in DMEM containing 10% fetal bovine serum (FBS). Then, the supernatant was aspirated and the cells were washed for three times with PBS. Subsequently, the cells were incubated with 100 μL of serum-free DMEM for 2 h, followed by induction with 100 μL 1 ×10^−7^ mmol/L insulin for 72 h. Next, the cells were incubated with 100 μL of LW-CKFH samples at various concentrations (prepared in DMEM medium) for an 24 h, as well as metformin was selected as a drug group, and DMEM medium without samples served as the blank group. The cell culture supernatant was collected to measure the glucose consumption using glucose colorimetric assay kit. Six replicate wells were used to test each sample (n = 6).

### 2.7. High Glucose and High-Fat Diet (HSHFD) Combined with STZ Induced T2DM Mice Model

Male Kunming strain SPF mice weighing 20.0 ± 0.2 g at 4 weeks of age were acquired from Shanghai Jieshijie Laboratory Animal Co., Ltd. (Animal License No. SCXK 2018-0004, Shanghai, China). The Shanghai Ocean University Animal Ethics Committee (SHOU-DW-2023-219) approved all animal experiments and procedures used in this study, and they were carried out in compliance with the rules and guidelines of the National Research Council’s Guide for the Care and Use of Laboratory Animals (IACUC No. 3590). Following one week of acclimatization to standard conditions (25 °C, 12 h light/dark cycle), all mice were randomly divided into six groups, with six mice per group. The control group received a standard diet, whereas the five experimental groups were fed a high-sugar high-fat diet (HSHFD) to establish the model. After a 4-week dietary intervention, all animals were fasted for 12 h with water provided ad libitum. Mice in the model group received intraperitoneal injections of 60 mg/kg STZ solution (prepared in 0.1 mol/L sodium citrate buffer) at 1-day intervals for a total of two injections. Normal group mice received an equal volume of sodium citrate buffer. Following the injections, the mice were fasted for 12 h, after which fasting blood glucose (FBG) levels were measured via tail vein sampling using a Roche ACCU-CHEK blood glucose meter (Shanghai, China) [18]. The T2DM mouse model was considered successfully established if FBG ≥ 11.1 mmol/L. After T2DM induction, mice were randomly divided into six groups (n = 6 per group) and treated via oral gavage for 4 weeks as follows: the normal control and T2DM model groups received saline; the positive control group received metformin (200 mg/kg·bw); and three treatment groups received LW-CKFH (1–3 kDa) at low (200 mg/kg·bw), medium (400 mg/kg·bw), and high (800 mg/kg·bw) doses. Throughout the intervention, body weight and fasting blood glucose levels were recorded every 7 days.

### 2.8. Oral Glucose Tolerance Test (OGTT)

Mice were given a glucose solution at a dose of 2.0 g/kg body weight following a 12 h fast following four weeks of medication administration. A blood glucose meter, marked G0 h, G0.5 h, G1 h, and G2 h, was then used to monitor blood glucose levels at 0 h, 0.5 h, 1 h, and 2 h. The area under the curve (AUC) for glucose tolerance was then computed using the following formula after the blood glucose curve was drawn.

(3) AUC=0.25×(G0 h+G0.5 h)+0.25×(G0 h+G1 h)+0.5×(G1 h+G2 h)

### 2.9. Serum Biochemical Parameter Assay

After a 4-week administration period, mice were fasted for 12 h. After the fasting period, the mice were anesthetized with ether, blood was drawn from their eyes, and they were euthanized by cervical dislocation. The blood was allowed to stand at 4 °C for 2 h, after which serum was obtained by centrifugation at 2500 rpm and 4 °C for 10 min. A panel of serum biomarkers, including GSP, INS, TC, TG, HDL-C, LDL-C, IL-2, and IL-6, was quantified. The homeostasis model assessment (HOMA) was used to evaluate pancreatic β-cell function and insulin resistance. Specifically, the following formulas were applied to the fasting glucose and insulin data to calculate HOMA of insulin resistance (HOMA-IR), insulin sensitivity (HOMA-IS), and β-cell function (HOMA-β) [18].

(4) $$\mathrm{HOMA}\text{-}\mathrm{IR}=\frac{FBG\times INS}{22.5}$$

(5) $$\mathrm{HOMA}\text{-}\mathrm{IS}=\frac{1}{HOMA-IR}$$

(6) $$\mathrm{HOMA}\text{-}\beta =20\times \frac{INS}{FBG-3.5}\times 100$$

### 2.10. Observation of Liver Tissue Pathology

Before being embedded in paraffin, mouse liver tissue was fixed in 4% paraformaldehyde for a full day. The hematoxylin and eosin (H&E) staining procedure was then used to generate slices that were 4 μm thick using a tissue microtome. Finally, the liver tissue structure and morphology were observed under a 400× magnification.

### 2.11. In Vitro Simulation Studies of the Gastrointestinal Digestive Process

The method for simulating gastrointestinal digestion in vitro described by Cai et al. [19] was slightly modified. A LW-CKFH solution (20 mg/mL) was prepared using blank simulated gastric juice, and the pH was adjusted to 2.0. After incubation at 37 °C for 10 min, 4 mL of simulated gastric juice was added. Following reaction at 37 °C and 120 rpm for 2 h, the mixture was heated in boiling water for 10 min to inactivate pepsin. After cooling, the pH of the solution was adjusted to 7.0, and it was incubated at 37 °C for 10 min. Then, 4 mL of simulated intestinal fluid was added, and the mixture was reacted at 37 °C and 120 rpm for 2 h, followed by boiling for 10 min. The supernatant obtained after centrifugation at 8000 rpm for 10 min constituted the gastrointestinal digest. In addition, a 20 mg/mL LW-CKFH solution was hydrolyzed under the above conditions for 4 h to simulate gastric and intestinal digestion, respectively. The experiment used an untreated LW-CKFH solution (20 mg/mL, pH 7.0, 25 °C) as the control group. No metal ions, food matrix, or gastrointestinal digestion were added to this solution, and its α-glucosidase inhibitory activity was defined as 100%. The α-glucosidase inhibitory activity of the gastrointestinal digestion supernatant was measured according to Method 2.4 (Formula (1)), and the retention rate of α-glucosidase inhibition in the gastrointestinal digestion supernatant was calculated as the ratio to the control group (refer to Formula (2)).

### 2.12. Analysis of the Processing Stability of LW-CKFH

An LW-CKFH solution (20 mg/mL) was prepared and subjected to treatment at different temperatures (25, 37, 50, 70, and 121 °C) and different pH values (2.0, 4.0, 7.0, 9.0, and 11.0 at 25 °C). It was also mixed with equal volumes of 2 mmol/L solutions of potassium chloride, calcium chloride, aluminum chloride, magnesium chloride, and ferric chloride and treated at 25 °C for 2 h. Immediately after heat treatment, the samples were cooled in an ice bath; all pH-treated samples were finally adjusted to 7.0 [19]. The α-glucosidase inhibition rate of the control group (20 mg/mL, 25 °C, pH 7.0, no metal ions) was set at 100%. The α-glucosidase inhibitory activity of the treated LW-CKFH was determined using the method described in Section 2.4. The α-glucosidase inhibition retention rate was calculated as the ratio of the activity of the treated LW-CKFH sample to that of the control group (Formula (2)).

### 2.13. α-Glucosidase Inhibitory Activity Analysis of LW-CKFH in Different Food Matrices

Twelve types of food were selected according to the “Chinese Dietary Guidelines (2022)” (http://dg.cnsoc.org/). Different food matrix solutions were prepared by homogenizing the recommended maximum daily intake of each food with 1500 mL of purified water. LW-CKFH was then added to each food matrix solution to a final concentration of 20 mg/mL, followed by in vitro gastrointestinal digestion as described in Section 2.11. After digestion, the mixture was boiled for 10 min and centrifuged at 8000 rpm for 10 min. The supernatant was collected, and its α-glucosidase inhibitory activity was determined according to the method described in Section 2.4. The experiment used an untreated LW-CKFH solution (20 mg/mL, pH 7.0, 25 °C)—that is, one to which no metal ions, food matrix, or gastrointestinal digestion had been added—as the control group, with its α-glucosidase inhibitory activity set at 100%. The α-glucosidase inhibition retention rate was calculated as the ratio of the measured activity to that of the control group (refer to Formula (2)).

### 2.14. Analysis of Glucose Content of Bread During Simulated Diet Digestion

The production of bread was prepared by the method of Devi et al. [20] with minor modifications. High-gluten wheat flour was first mixed with 1.2% (*w*/*w*) dry yeast, 1.2% (*w*/*w*) salt, 1 egg, 30% (*w*/*w*) cow milk and 4.8% (*w*/*w*) butter. Subsequently, 1.2% (*v*/*w*) LW-CKFH solution was added to prepare dough. The bread was baked for 35 min under upper heat at 210 °C and lower heat at 200 °C. Simulated saliva, gastric fluid, and intestinal fluid were prepared by modifying the method described by Minekus et al. [21]. The 5 g of bread was homogenized with 15 mL of simulated saliva, then incubated at 37 °C for 2 min under continuous shaking at 120 rpm. After adjusting the pH of mixture solution to 2.0, 30 mL of simulated gastric juice were added and kept reaction at 120 rpm for 2 h at 37 °C, After adjusting the pH of mixture solution to 7.0, 15 mL of simulated intestinal fluid was added and incubated. for 2 h at 37 °C. From intestinal digestion, 2 mL mixture solution was collected at 0 min, 15 min, 30 min, 60 min, and 120 min, respectively, and immediately boiled for 10 min to inactivate the enzyme. Finally, the glucose content in the reaction solution at different reaction times was determined using an a-glucose colorimetric assay kit, with bread without LW-CKFH serving as the blank control.

### 2.15. Identification and Screening of CKF Polypeptides

The CKF oligopeptide fraction F2 was analyzed by LC-MS/MS. The air-dried peptide sample was redissolved in a 0.1% formic acid-water solution. Separation was performed using the EASY-nLC1200 Nano-HPLC system (Ultimate 3000, Thermo Scientific, Waltham, MA, USA). Mobile phase A consisted of a 0.1% formic acid-water solution, and mobile phase B consisted of a 0.1% formic acid-acetonitrile solution. The Trap column (PepMap 100 C18, 3 μm, 75 μL × 2 cm, Agilent Technologies, Santa Clara, CA, USA) was equilibrated with 100% mobile phase A. Samples were loaded via an autosampler and adsorbed onto a Trap column, followed by separation on an Analysis column (PepMap C18 2 μm, 75 μm × 25 mm, Agilent Technologies) at a flow rate of 750 nL/min. A 30 min mobile phase gradient with blank solvent was used to flush the system between samples. After separation by capillary HPLC, the proteolytic products were analyzed using an Obitrap Fusion Lumos mass spectrometer (Q-Exactive, ThermoFisher, USA) in DDA mode. Detection method: Mass spectrometry scanning was performed in data-dependent acquisition (DDA) mode, with a first-stage mass spectrometry resolution of 60,000, a scan range of 350–1500 *m*/*z*, and a maximum injection time of 118 ms. Each DDA cycle lasted 2 s, with a maximum injection time of 22 ms for MS/MS ions. The collision chamber energy (High-Energy Collision-Induced Dissociation, HCD) was set to 30% for all precursor ions, and the dynamic exclusion time was set to 35 s. Raw data files from mass spectrometry acquisition were processed and analyzed using PEAKS Studio 8.5 (Bioinformatics Solutions Inc., Waterloo, ON, Canada). The identification database used was the UniProt-Cocos nucifera (Coconut palm)-UP000797356_13894 protein database. The peptide sequences and molecular weights were obtained from liquid chromatography–mass spectrometry (LC-MS/MS) results. Peptide Ranker (http://distilldeep.ucd.ie/PeptideRanker/, accessed on 10 February 2025) was used to rank the predicted bioactivity scores of the peptides, and those with a score of 0.5 were selected. ToxinPred (https://webs.iiitd.edu.in/raghava/toxinpred/, accessed on 11 February 2025) was used to predict the potential toxicity of the peptide sequences. Allergenicity was predicted using AllerTOP v.2.1 (https://www.ddg-pharmfac.net/AllerTOP/, accessed on 11 February 2025). Only non-toxic and non-allergenic peptides were retained for further screening. PepDraw was used to predict the physicochemical properties of the peptide sequences online, including hydrophobicity, net charge, and isoelectric point [22].

### 2.16. Molecular Docking

Exploring the binding modes of peptides with α-glucosidase using AutoDock Vina version 1.1.2 [23]. The crystal structure of α-glucosidase was obtained from the PDB database (https://www.rcsb.org/) (PDB ID: 3WY1). In PyMOL 2.3.0 (Delano Science LLC), the protein structures were dehydrated, the original ligands were removed, and the structures were preprocessed. The processed protein structures were then imported into AutoDock Vina to complete hydrogenation, charge calculations, charge distribution, and atom type assignment. The docking region settings were as follows: center x = −6.665; center y = −12.615; center z = 21.514. The grid box size was 80 × 80 × 80 Å, with a spacing of 0.375 Å per grid; all other parameters remain at their default values.

### 2.17. Statistical Analysis

All experiments were repeated at least three times, and data are presented as mean ± standard deviation. Statistical analysis was performed using IBM SPSS 26 software (IBM, Armonk, NY, USA). One-way ANOVA followed by Duncan’s multiple-range test was applied to assess significant differences among groups (*p* < 0.05, significant difference). All figures were generated using Origin 2024 software (OriginLab Corporation, Northampton, MA, USA). All schematics were generated using the Generic Diagramming Platform (GDP, https://www.BioGDP.com) [24].

## 3. Results

### 3.1. Preparation of LW-CKFH with High α-Glucosidase Inhibitory Activity

**Figure 1:**
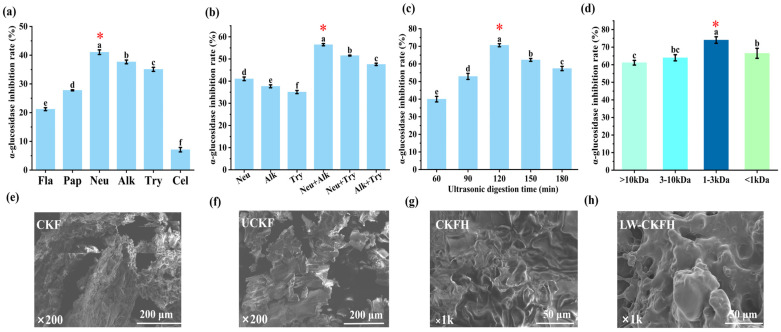
Preparation of LW-CKFH with high α-glucosidase inhibitory activity. (**a**) Inhibition rates of α-glucosidase by different proteases; (**b**) inhibition rates of α-glucosidase by a protease mixture; (**c**) effect of ultrasonication time on the inhibition rate of α-glucosidase; (**d**) inhibition rates of α-glucosidase by ultrafiltration fractions with four different molecular weights; (**e**) scanning electron microscope (SEM) image of CKF; (**f**) SEM image of CKF after ultrasonic treatment; (**g**) SEM image of CKFH; and (**h**) SEM image of LW-CKFH. The abbreviations in the figure represent: flavor protease (Fla), papain (Pap), neutral protease (Neu), alkaline protease (Alk), trypsin (Try), and cellulase (Cel). Different letters indicate significant differences (*p* < 0.05). * indicates the component with the best result, highlighted for clear visualization.

To prepare CKFH with high α-glucosidase inhibitory activity, the effects of different protease types on the α-glucosidase inhibitory activity of CKF were investigated. First, six common proteases were used for enzymatic hydrolysis. CKFH obtained with neutral protease exhibited the highest α-glucosidase inhibitory activity (41.03%) (Figure 1a). Neutral proteases are endopeptidases with broad cleavage specificity; they preferentially hydrolyze the C-terminal peptide bonds of hydrophobic amino acids, facilitating entry into the enzyme active pocket and the formation of hydrophobic interactions, thereby enhancing inhibitory activity [8]. According to Yang et al. [25], combined enzymatic hydrolysis significantly enhanced the α-glucosidase inhibitory activity of peanut meal protein hydrolysate. Therefore, the effect of combined enzymatic hydrolysis on CKFH activity was further investigated in this study. The combined hydrolysis of neutral protease and alkaline protease increased the inhibitory activity of CKFH against α-glucosidase to 56.46%, which was 15% higher than that achieved with neutral protease alone (Figure 1b). This is mainly attributed to the different hydrolysis specificities of the two enzymes, allowing their combination to achieve higher hydrolysis efficiency [26]. To further enhance the α-glucosidase inhibitory activity of CKFH, the effect of ultrasonic treatment on CKFH activity was investigated. CKFH prepared by ultrasonic-assisted enzymatic hydrolysis for 120 min exhibited an α-glucosidase inhibitory activity of 70.14% (Figure 1c), representing an increase of 19.5% compared with the initial level. The influence of ultrasonic treatment on the microstructure of CKF was observed using scanning electron microscopy (SEM). Obviously, the ultrasonic treatment significantly reduced the volume of CKF aggregates, and the large sheet-like structures were broken down into smaller fragments (Figure 1e,f). Thus, ultrasonic treatment for 120 min significantly disrupted the CKF structure, exposing hydrophobic groups within the protein, thereby enhancing the hydrolysis efficiency. This disruption might be attributed to the mechanical force and cavitation effects generated by ultrasound, which disrupted the complex triple-helix conformation of the protein [27]. In summary, we optimized and established the optimal enzymatic hydrolysis process for CKFH, which employed an ultrasound-assisted dual-enzyme hydrolysis method using neutral and alkaline proteases at an enzyme concentration of 8000 U/g, with each hydrolysis step lasting 2 h.

Although CKFH exhibited good α-glucosidase inhibitory activity, it also contained substantial impurities with low or no activity. Therefore, CKFH was performed to ultrafiltrate, yielding four active fractions. The LW-CKFH fraction with a molecular weight of 1–3 kDa exhibited the highest α-glucosidase inhibitory activity by 74.49% (Figure 1d). SEM results revealed that the CKFH fraction exhibited a smooth, plate-like structure on the surface (Figure 1g), whereas the LW-CKFH fraction displayed a loose, porous structure (Figure 1h). The structural differences between the two fractions suggested that the porous structure of the LW-CKFH fraction facilitated its binding to α-glucosidase and enhanced its inhibitory activity. Previous studies showed that low-molecular-weight bioactive peptides could more effectively inhibit α-glucosidase activity through competitive binding to its active site [28,29], which was helpful to slow down the digestion of carbohydrates and lower blood glucose levels. Based on these findings, the present study investigated the hypoglycemic efficacy and mechanism of action of LW-CKFH using in vitro cell models and in vivo animal models.

### 3.2. The Glucose Metabolism Effects of LW-CKFH Based on IR-HepG2 Cell Model

**Figure 2:**
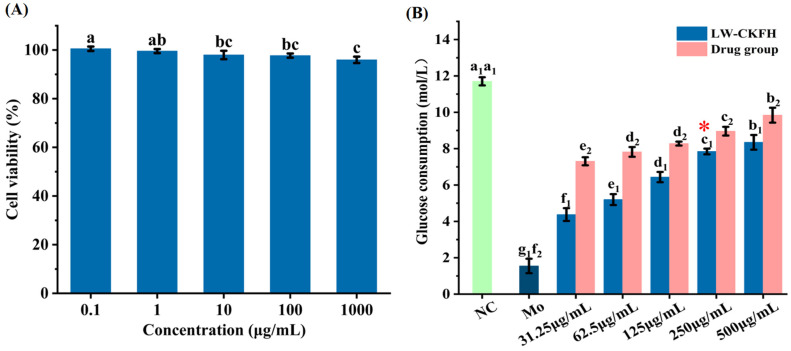
Analysis of the glucose metabolism-improving effects of LW-CKFH using the IR-HepG2 cell model. (**A**) The effect of LW-CKFH on cell viability; (**B**) the effect of LW-CKFH on glucose consumption of IR-HepG2 cells. Different letters indicate significant differences (*p* < 0.05). * indicates the component with the best result, highlighted for clear visualization.

Good biosafety is essential for the development of novel hypoglycemic agents. Therefore, the biosafety of LW-CKFH was evaluated using HepG2 cells. As shown in Figure 2A, the viability of HepG2 cells remained above 95% after 24 h of treatment with LW-CKFH at concentrations ranging from 0.1 to 1000 μg/mL, indicating that LW-CKFH had no cytotoxicity over a wide concentration range and possessed good biosafety. Insulin resistance (IR) is a key factor leading to impaired glucose metabolism and the development of T2DM [30]. The liver is one of the key organs involved in glucose metabolism. Therefore, an IR-HepG2 cell model was used to investigate the effects of LW-CKFH on cellular glucose metabolism. As shown in Figure 2B, glucose consumption in the IR model group was decreased by 86.21% compared with the normal control group. However, after LW-CKFH intervention, glucose consumption increased in a dose-dependent manner. Notably, 250 μg/mL of LW-CKFH could restore glucose consumption to 95.4% of that achieved with an equivalent dose of metformin. These results indicated that LW-CKFH effectively increases glucose uptake, enhances glucose metabolism in IR-HepG2 cells, and reduced insulin resistance.

### 3.3. The Regulatory Effect of LW-CKFH on Glucose Homeostasis in T2DM Mice

#### 3.3.1. The Effect of LW-CKFH on Improving Glucose Metabolic Disorders in T2DM Mice

**Figure 3:**
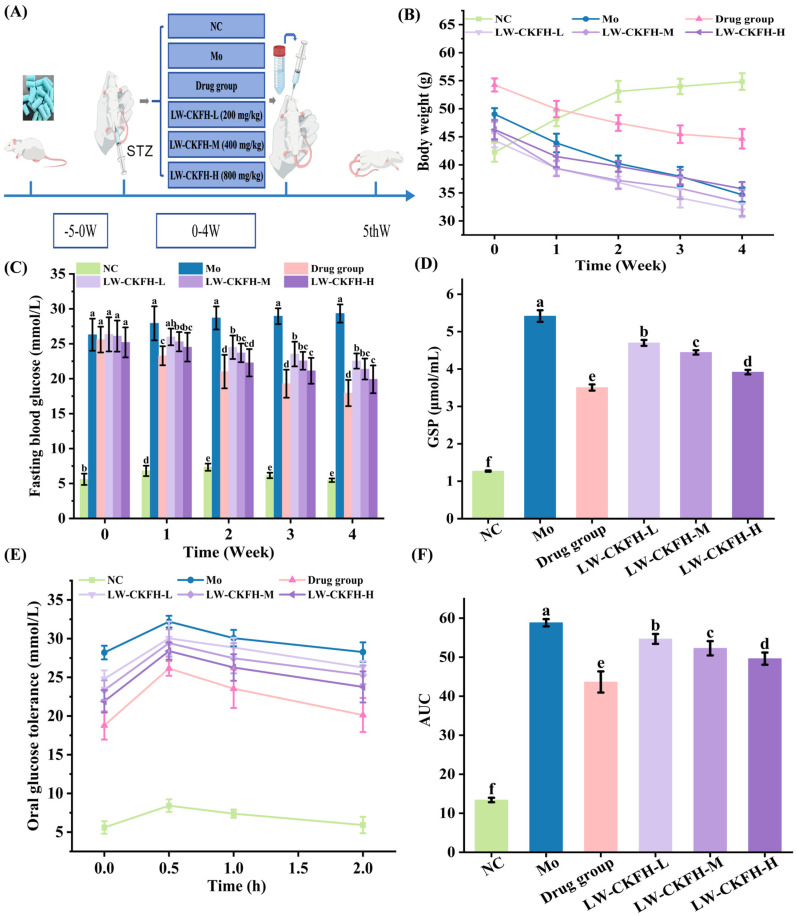
The effect of LW-CKFH on improving glucose metabolic disorders in mice with T2DM. (**A**) HSHFD/STZ-induced mice model and treatment; (**B**) body weight; (**C**) fasting blood glucose; (**D**) GSP; (**E**) OGTT, and (**F**) AUC. HSHFD/STZ-induced mice were divided into model group, high-dose group, medium-dose group, low-dose group, and drug metformin group (Mo, LW-CKFH-H, LW-CKFH-M, LW-CKFH-L, and drug group). Different letters indicate significant differences (*p* < 0.05).

Given the promising hypoglycemic potential of LW-CKFH at the cellular level, an HSHFD-STZ-induced T2DM mouse model was established, and treated with LW-CKFH for 4 weeks to evaluate its in vivo hypoglycemic efficacy and regulatory effects on glucose homeostasis (Figure 3A). Compared with the normal control group, all mice in the T2DM model group exhibited weight gain during the induction period (Figure 3B), and fasting blood glucose (FBG) levels exceeded 11.1 mmol/L (Figure 3C), confirming the successful construction of the T2DM mouse model. Notably, all T2DM mice showed persistent weight loss, while the model group exhibited the most pronounced reduction (25.8%). This was primarily attributed to STZ-induced impairment of insulin secretion in pancreatic β-cells, which prevented normal glucose metabolism for energy production and led to the catabolism of proteins in muscle tissue. After 4 weeks of intervention, the rate of weight loss was significantly reduced in both the metformin and LW-CKFH groups, with decreasing of 17.7% and 22.8%, respectively. Furthermore, LW-CKFH intervention effectively reduced FBG levels in T2DM mice (Figure 3C). Compared with the model group, FBG levels in the metformin, LW-CKFH-H, LW-CKFH-M and LW-CKFH-L groups decreased by 38.7%, 32.0%, 27.0%, and 23.1%, respectively. Obviously, the improving effect of LW-CKFH on FBG showed a dose-dependent pattern. The efficacy of the high-dose group was comparable to that of the clinical drug metformin. These results indicated that LW-CKFH intervention could improve glucose metabolism in T2DM mice. Glycated serum protein (GSP) is a product formed by the reaction between serum proteins and glucose in the blood. Due to persistently elevated blood glucose levels in patients with type 2 diabetes, their GSP levels might increase significantly [31]. As shown in Figure 3D, serum GSP levels in the model group were significantly elevated, reaching 326.8% of those in the normal control group, indicating severe glucose metabolism disorders in mice with type 2 diabetes induced by a high-fat diet combined with STZ. Notably, after LW-CKFH intervention, GSP levels in T2DM mice were significantly reduced (*p* < 0.05). In the LW-CKFH-H group, GSP level were significantly reduced by 27.7% (*p* < 0.05), reaching 72.32% of those in the metformin group. These results indicated that LW-CKFH intervention effectively lowered blood glucose levels, thereby ameliorated glucose metabolism disorders in T2DM mice. Further analysis showed that LW-CKFH intervention effectively reduced the postprandial blood glucose peak in T2DM mice after oral glucose administration (Figure 3E). Among the treatment groups, the high-dose LW-CKFH intervention demonstrated the best results. AUC calculations revealed that LW-CKFH exerted a dose-dependent effect on the AUC in T2DM mice, with lower AUC values observed at higher concentrations (Figure 3F). The high-dose LW-CKFH group reduced the AUC value by 15.67%, approaching that of the metformin group (25.8%). This indicated that LW-CKFH effectively improved glucose metabolism disorders and lowered blood glucose levels in T2DM mice.

#### 3.3.2. The Improvement Effect of LW-CKFH on the Insulin Level in T2DM Mice

**Figure 4:**
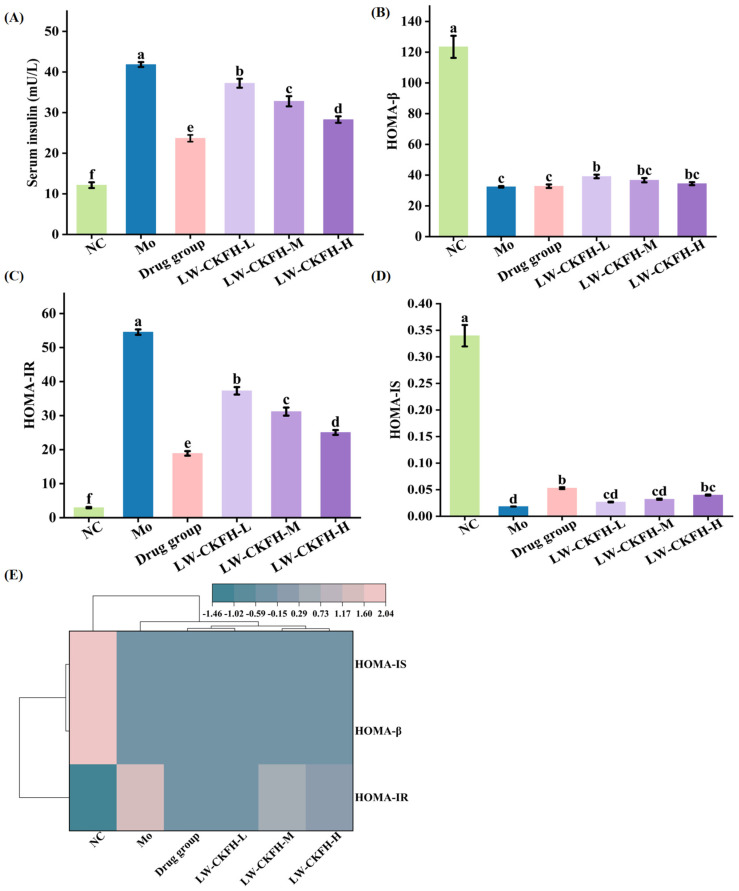
The improvement effect of LW-CKFH on the insulin level in T2DM mice. (**A**) Serum insulin levels; (**B**) HOMA-β; (**C**) HOMA-IR; (**D**) HOMA-IS, and (**E**) Cluster heat map. Different letters indicate significant differences (*p* < 0.05).

Insulin is the only hormone that regulates cellular glucose uptake and metabolism. However, T2DM leads to an increasing of insulin resistance and decreasing of insulin sensitivity, which can result in reduced insulin efficacy and consequently elevate serum insulin levels [32]. As shown in Figure 4A, serum insulin levels in the model group were significantly increased by 73.6% compared with the normal control group. Notably, after LW-CKFH intervention, serum insulin concentrations in T2DM mice were significantly reduced (*p* < 0.05), with the lowest levels observed in the LW-CKFH-H group (a 32.41% decrease). To further elucidate the mechanism by which LW-CKFH improved insulin sensitivity in T2DM mice, HOMA-β, HOMA-IR, and HOMA-IS were calculated using the HOMA model. The results showed that compared with the model group, HOMA-β values in the metformin group and all LW-CKFH dose groups were not significantly increased (*p* > 0.05) (Figure 4B). This indicated that neither LW-CKFH nor metformin had a significant effect on promoting insulin secretion in T2DM mice. Notably, HOMA-IR values in the T2DM model group were significantly increased, suggesting that T2DM mice exhibited severe IR (Figure 4C). Following LW-CKFH intervention, HOMA-IR decreased significantly in a dose-dependent manner. Among the groups, the LW-CKFH-H group showed the most pronounced decrease (53.72%), which was closest to that observed in the metformin group (65.36%). This indicated that LW-CKFH intervention significantly reduced IR levels in T2DM mice, with an improvement reaching 75.43% of that achieved by metformin. The interventions of metformin and high-dose LW-CKFH only restored the HOMA-IS levels of T2DM mice to 15.59% and 11.76% of the normal control group, respectively, and there was no significant difference between the two (*p* > 0.025) (Figure 4D). This suggested that LW-CKFH intervention had a limited effect on improving insulin sensitivity in T2DM mice. To further clarify the primary mechanisms by which LW-CKFH improved insulin action in T2DM mice, an agglomerative heatmap analysis was performed. As shown in Figure 4E, HOMA-IR was clustered separately, and all T2DM mice were grouped into a single cluster, indicating that the pathological characteristics of the T2DM mouse model established in this study were primarily characterized by IR. This suggested that metformin and LW-CKFH primarily improved insulin action and enhanced insulin sensitivity in T2DM mice by reducing IR levels. Notably, the metformin group and the LW-CKFH-H group clustered together, indicating that the effect of LW-CKFH-H intervention on improving IR levels in T2DM mice was similar to that of metformin.

#### 3.3.3. Improvement of LW-CKFH on Lipid Metabolism Disorder in T2DM Mice

**Figure 5:**
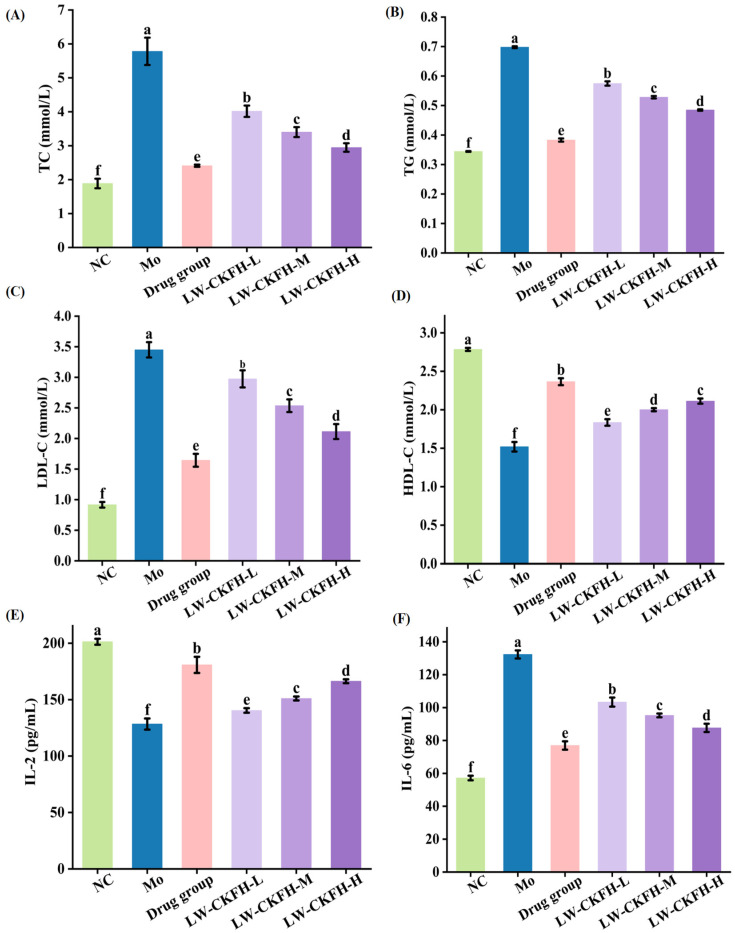
The effects of LW-CKFH on lipid metabolism and inflammatory responses in T2DM mice. (**A**) TC; (**B**) TG; (**C**) LDL-C; (**D**) HDL-C; (**E**) IL-2, and (**F**) IL-6. Different letters indicate significant differences (*p* < 0.05).

In diabetic patients, once glucose metabolism is disrupted and insulin resistance develops, liver glucose metabolism becomes impaired, often accompanied by lipid metabolism abnormalities. Specifically, this manifests as reduced HDL-C synthesis, which in turn further exacerbates hepatic insulin resistance, creating a vicious cycle. Clinical studies have confirmed that fluctuations in TG, TC, HDL-C, and LDL-C levels are closely associated with the onset and progression of cardiovascular disease and atherosclerosis. Consequently, prolonged lipid metabolism disorders significantly increased the risk of cardiovascular disease [33]. Therefore, the effects of each treatment on TG, TC, LDL-C, and HDL-C levels in T2DM mice were investigated. As shown in Figure 5A–D, compared with the normal control group, HFD/STZ-induced mice exhibited significant dyslipidemia, characterized by elevated levels of TC, TG, and LDL-C, and reduced HDL-C (*p* < 0.05). After 4 weeks of treatment, lipid metabolism disorders were significantly improved in the LW-CKFH and metformin groups. Among these, the LW-CKFH-H group showed the best results, reducing TC, TG, and LDL-C by 49%, 34.8%, and 38.8%, respectively. In addition, HDL-C levels in T2DM mice in the LW-CKFH-H group increased significantly after intervention, with a dose-dependent rise of 27.96%. These results indicated that LW-CKFH administration not only alleviated hyperglycemia but also restored lipid homeostasis, thereby contributing to the recovery of hepatic glucose metabolism.

#### 3.3.4. The Improvement of LW-CKFH on Inflammatory Response in T2DM Mice

T2DM is characterized by severe disturbances in glucose and lipid metabolism, which can further trigger oxidative stress and lipid peroxidation [34]. These metabolic abnormalities lead to the production of excessive reactive oxygen species (ROS) and malondialdehyde (MDA), thereby inducing inflammatory responses. Consequently, this results in increased production of the pro-inflammatory cytokine IL-6 and suppression of the anti-inflammatory cytokine IL-2. As shown in Figure 5E,F, compared with the normal control group, IL-2 levels in the model group were significantly reduced by 36.22% (*p* < 0.05), while IL-6 levels were significantly increased by 53.62% (*p* < 0.05). These findings indicated that disrupted glucose and lipid metabolism led to a marked inflammatory response in T2DM mice. Following LW-CKFH intervention, IL-2 levels in T2DM mice increased significantly while IL-6 levels decreased significantly (*p* < 0.05), with both effects exhibiting a dose-dependent relationship. This suggested that LW-CKFH intervention effectively promoted the production of anti-inflammatory cytokines and suppressed the production of pro-inflammatory cytokines, thereby alleviating the inflammatory response. Notably, high-dose LW-CKFH intervention achieved 91.98% and 87.8% of the effects of metformin in promoting IL-2 and inhibiting IL-6, respectively. This indicated that the efficacy of LW-CKFH in improving the inflammatory response in T2DM mice was comparable to that of the clinical drug metformin. These results confirmed the beneficial effects of LW-CKFH in improving glucose and lipid metabolism disorders and alleviating inflammatory responses in T2DM mice.

#### 3.3.5. The Reparative Effects of LW-CKFH on Hepatocyte Damage in T2DM Mice

**Figure 6:**
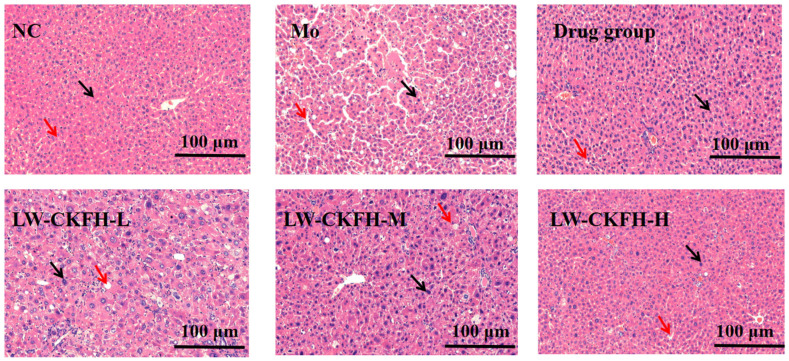
The reparative effects of LW-CKFH on hepatocyte damage in T2DM mice. Histological observation of liver tissue stained with H&E (400×). Black arrow: hepatocyte structure; red arrow: fatty infiltration (or cytoplasmic vacuolization).

Oxidative stress, lipid peroxidation, and inflammatory responses induced by T2DM can lead to the excessive accumulation of harmful substances such as ROS, MDA, and IL-6 [35]. These harmful substances can damage protein and DNA structures, thereby causing hepatocyte damage [36], which further exacerbating insulin resistance and metabolic disorders related to sugar and lipids. As shown in Figure 6, the model group exhibited fewer hepatocytes, marked cytoplasmic vacuolization, and inflammatory cell infiltration. With increasing doses of LW-CKFH, the structure of hepatocytes (black line) gradually became clearer, swelling decreased, and the cells were arranged more tightly. Cytoplasmic vacuolization was reduced, and fatty infiltration (red line) also improved significantly. Among the groups, the hepatic cell status in the metformin group and the LW-CKFH-H group was most similar to that of the control group, indicating that these two groups exhibited the best intervention effects. In summary, LW-CKFH exerted a dose-dependent reparative effect on hepatocyte damage in T2DM mice.

#### 3.3.6. The Main Regulatory Pathways of LW-CKFH in Blood Glucose Homeostasis of T2DM Mice

A summary of the effects of LW-CKFH on T2DM mice revealed that after 4 weeks of intervention, high-dose LW-CKFH (800 mg/kg·bw) exerted a regulatory effect on blood glucose homeostasis comparable to that of metformin (200 mg/kg·bw). LW-CKFH-H treatment reduced serum GSP levels by 27.7% (*p* < 0.05) and lowered FBG levels by 32%, indicating a sustained hypoglycemic effect. Furthermore, LW-CKFH-H effectively improved glucose tolerance, resulting in a significant 15.92% reduction in postprandial blood glucose levels. More importantly, the LW-CKFH-H treatment group exhibited the lowest serum insulin levels (a 32.41% reduction), suggesting enhanced insulin sensitivity and effective blood glucose control even with reduced insulin dosage. This study found that the HOMA-β profiles in the metformin and LW-CKFH intervention groups were similar to those in the model group, indicating that neither treatment improved insulin secretion. However, LW-CKFH-H intervention significantly reduced HOMA-IR in T2DM mice and increased HOMA-IS levels by 53.72% and 11.76%, respectively, approaching the reductions and increases observed with metformin (65.36% and 15.59%, respectively). These results indicated that LW-CKFH effectively improved glucose metabolism disorders in T2DM mice. Its mechanism of action was attributed to enhancing insulin sensitivity and promoting glucose uptake and utilization by peripheral tissues, thereby achieving effective blood glucose control.

**Figure 7:**
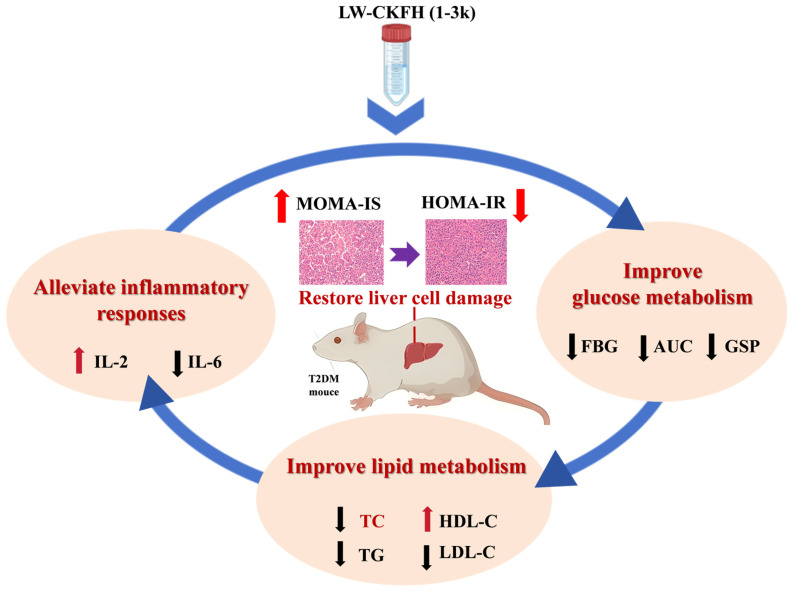
The main regulatory pathways of LW-CKFH in blood glucose homeostasis of T2DM mice.

As the central organ for glucose and lipid metabolism, the liver plays a critical role in alleviating glucose and lipid metabolism disorders in T2DM patients [37]. It was hypothesized that LW-CKFH-H intervention primarily improved glucolipid metabolism disorders and chronic inflammation in T2DM mice by reducing hepatic IR. The results showed that LW-CKFH-H significantly reversed T2DM-induced lipid metabolism abnormalities. Specifically, it significantly reduced TC, TG, and LDL-C levels by 49%, 34.8%, and 38.8%, respectively, while increasing HDL-C levels by 27.96%. This improvement mechanism was consistent with the findings of Ma et al. [38], who reported that ameliorating IR helped suppress the formation of TC and LDL-C. Furthermore, improvements in glucose and lipid metabolism were often closely associated with the alleviation of chronic inflammation. In this study, LW-CKFH intervention effectively downregulated the pro-inflammatory cytokine IL-6 (to 65.24% of normal levels) and upregulated the anti-inflammatory cytokine IL-2 (to 82.26% of normal levels). This finding was consistent with the results reported by Li et al. [39], who demonstrated that sweet triterpenoid glycosides derived from *Paulownia* leaves could improve glucose and lipid metabolism in T2DM mice, thereby helping to alleviate inflammatory responses. Thus, LW-CKFH might mitigate inflammatory responses by improving dysregulated glucose and lipid metabolism. The 4-week LW-CKFH intervention significantly reduced FBG levels and improved glucose tolerance in T2DM mice, demonstrating a favorable hypoglycemic effect. Therefore, these results suggested that LW-CKFH regulated glucose homeostasis in T2DM mice primarily by alleviating hepatic insulin resistance through the repair of hepatocyte damage, thereby promoting glucose metabolism. This mechanism further improved lipid metabolism abnormalities and helped alleviate chronic inflammation. Ultimately, this regulatory pathway enhanced insulin sensitivity, thereby regulating glucose homeostasis in T2DM mice (Figure 7).

### 3.4. Feasibility of LW-CKFH in the Application of Hypoglycemic Food

#### 3.4.1. Processing Stability of LW-CKFH

**Figure 8:**
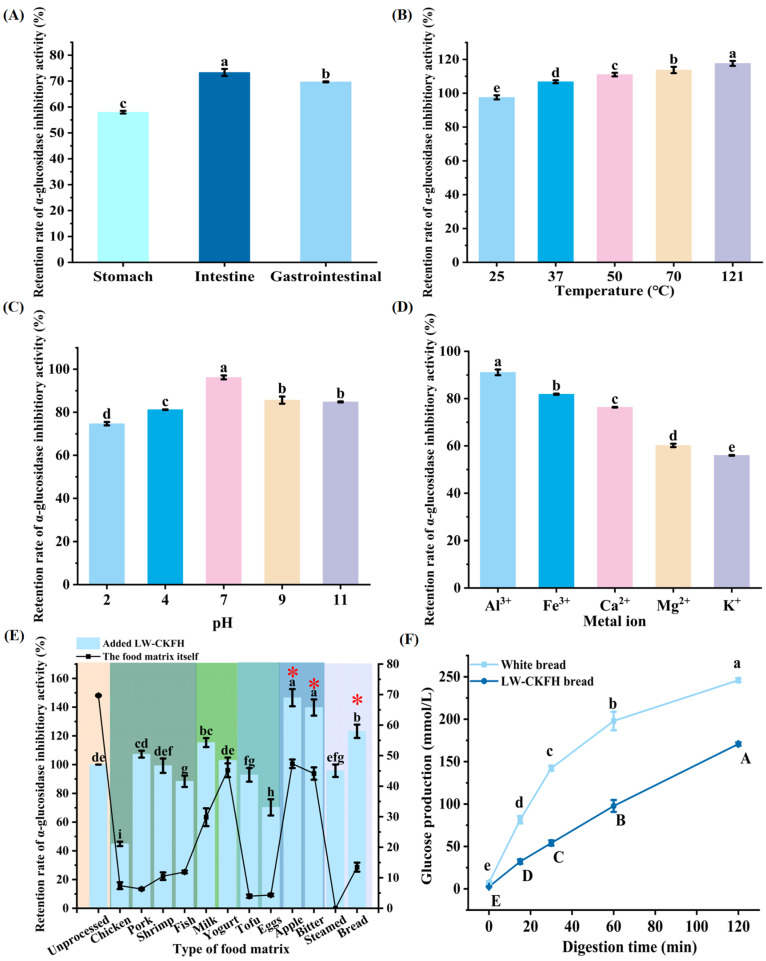
Feasibility of LW-CKFH in the application of hypoglycemic food. (**A**) The retention rate of α-glucosidase inhibitory activity after gastrointestinal digestion; (**B**) the retention rate of α-glucosidase inhibitory activity at different temperatures; (**C**) the retention rate of α-glucosidase inhibitory activity under different pH conditions; (**D**) the retention rate of α-glucosidase inhibitory activity in different metal ion solutions; (**E**) the retention rate of α-glucosidase inhibitory activity in different food matrices, and (**F**) glucose production in LW-CKFH bread after in vitro digestion. Different letters indicate significant differences (*p* < 0.05). * indicates the component with the best result, highlighted for clear visualization.

To be absorbed and utilized, oral active peptides must withstand the extreme acidic and alkaline environments and enzymatic action in the gastrointestinal tract [40]. In vitro simulated gastrointestinal digestion results showed that after 2 h of digestion with simulated gastric and intestinal fluids, respectively, the retention rates of α-glucosidase inhibitory activity for LW-CKFH were 57.99% and 73.33% (Figure 8A). Notably, after 4 h of continuous simulated gastrointestinal digestion, the activity retention rate of LW-CKFH was 69.69%, indicating that it possessed good resistance to gastrointestinal digestion. Heat treatment is a common sterilization method in food processing. Therefore, the thermal stability of LW-CKFH was investigated. After 2 h of heating at temperatures ranging from 37 to 121 °C, its α-glucosidase inhibitory activity was proportional to temperature and remained higher than that at 25 °C (Figure 8B), indicating that LW-CKFH was heat-stable. The increase in activity was speculated to be related to the Maillard reaction between peptides and reducing sugars promoted by high temperatures, resulting in the formation of glycosylated products with stronger inhibitory activity. This phenomenon was consistent with the findings of Rivero-Pino, who reported that the ACE inhibitory activity of sardine protein hydrolysates increased by more than 50% after heat treatment [41].

Given the need to add food additives, flavorings, and other auxiliary ingredients during food production and processing, the effects of pH and metal ions on the activity of LW-CKFH were further investigated. As shown in Figure 8C, LW-CKFH exhibited optimal inhibitory activity under neutral conditions (pH 7.0). Under strongly acidic (pH 2.0) and strongly alkaline (pH 11.0) conditions, the retention rates of α-glucosidase inhibitory activity for LW-CKFH remained above 75%, indicating good pH stability. However, its activity was lower under strongly acidic conditions than under strongly alkaline conditions, suggesting that it was more suitable for dissolution in neutral or alkaline systems for addition to food products. The effects of different metal ions on the activity of LW-CKFH were shown in Figure 8D. Al^3+^ ions had the least effect on the α-glucosidase inhibitory activity of LW-CKFH, with an activity retention rate of 91.1%, followed by Fe^3+^ and Ca^2+^ (>70%). Mg^2+^ and K^+^ were found to have a significant impact on the α-glucosidase inhibitory activity of LW-CKFH. In the presence of K^+^, the inhibitory activity decreased to 55.99%. Nawaz et al. reported that during food processing, trace metal ions might act as strong oxidizing agents to affect the oxidative modification of specific amino acid residues on polypeptide chains, thereby altering the chemical composition and biological activity of proteins and peptides [42]. Therefore, when using LW-CKFH, solutions containing Mg^2+^ and K^+^ should be avoided where possible.

#### 3.4.2. The Effect of Food Matrix on LW-CKFH Activity

To investigate the effect of food matrices on the activity of LW-CKFH, we referred to dietary guidelines and selected 12 common food raw materials such as fish, milk, eggs, apples, and steamed buns to prepare different food matrix solutions. Then, we added LW-CKFH to various food matrices and conducted simulated gastrointestinal digestion. As shown in Figure 8E, after gastrointestinal digestion, apples and bitter melon retained the highest levels of their inherent α-glucosidase inhibitory activity, at 47% and 44%, respectively. The activity retention rates after LW-CKFH addition were also the highest by 146.58% and 139.75%, indicating a synergistic effect between the two. Similarly, yogurt, milk and bread exhibited high levels of activity retention on their own, and their activity retention rates also showed a synergistic effect in the LW-CKFH co-system. However, it was observed that the α-glucosidase inhibitory activity decreased in the co-system of LW-CKFH with food matrices such as meat, seafood, tofu and eggs, indicating that these food matrices could interfere with the activity of LW-CKFH. This might be attributed to the preferential degradation of the high-protein matrix by proteases during simulated digestion [43]. In this process, the degradation products are competitively bound to LW-CKFH through non-specific adsorption, thereby interfering with its binding to α-glucosidase. In conclusion, LW-CKFH was more suitable for being added to food items such as fruits and vegetables, dairy products and bread to develop functional foods with a low glycemic index (GI) or hypoglycemic effects.

#### 3.4.3. Effect of LW-CKFH on the Glucose Release Rate of Bread During Simulated Diet Digestion

**Figure 9:**
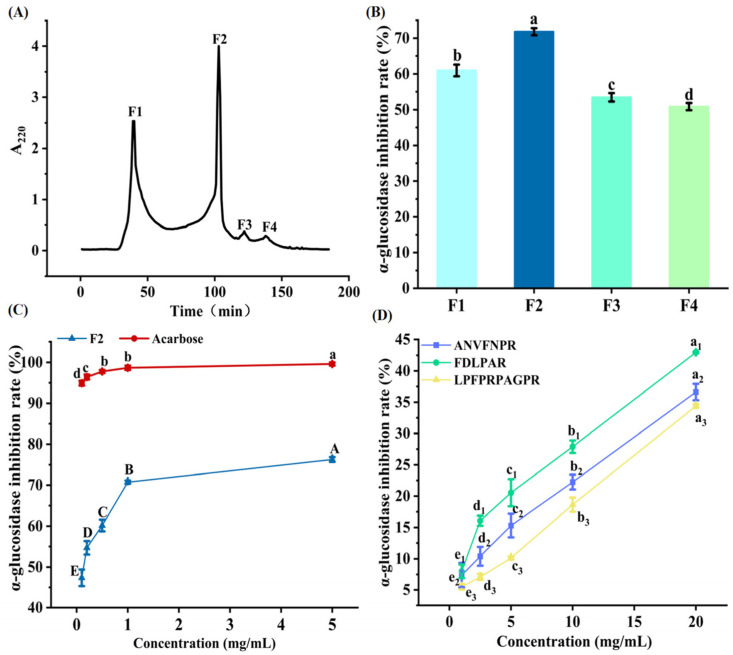

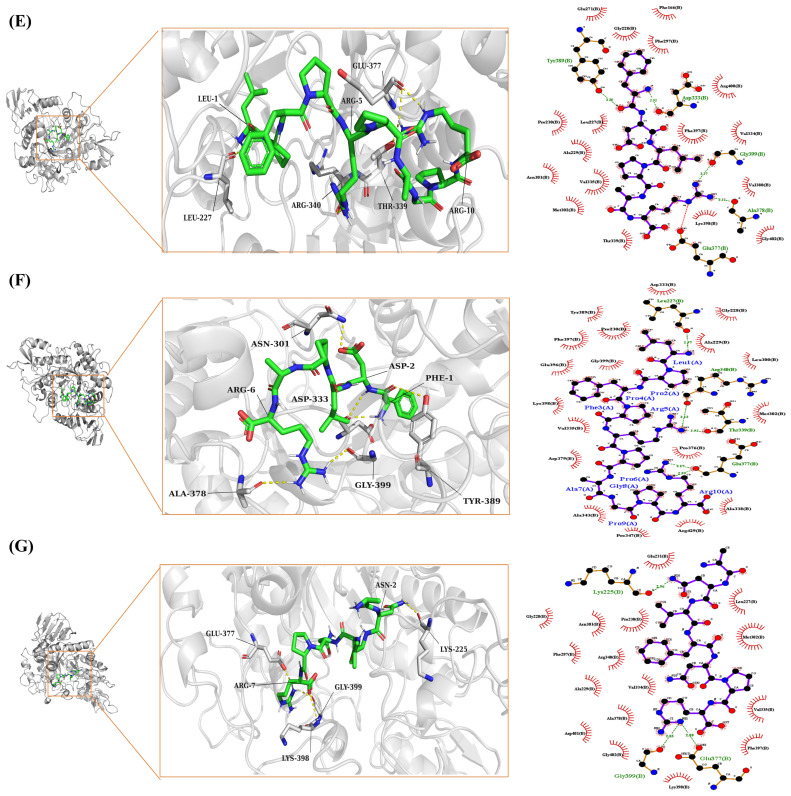
Activity evaluation and binding analysis of α-Glucosidase inhibitory peptides from CKF. (**A**) Sephadex G-25 gel filtration chromatography; (**B**) in vitro α-glucosidase inhibitory activity of gel-chromatographically separated fractions; (**C**) α-glucosidase inhibition rates of acarbose and F2 at different concentrations; (**D**) inhibition rates of α-glucosidase activity by synthetic peptides; (**E**) molecular docking results of FDLPAR with α-glucosidase; (**F**) molecular docking results of LPFPRPAGPR with α-glucosidase; (**G**) molecular docking results of ANVFNPR with α-glucosidase. Different letters indicate significant differences (*p* < 0.05).

To evaluate the role of LW-CKFH in control starch hydrolysis speed in food, the glucose release kinetics of bread added with LW-CKFH during simulated diet digestion was analyzed. After the bread was digested for 2 min simulated saliva and 2 h simulated gastric juice, simulated intestinal juice was added, and samples at different digestion times were collected to measure glucose content. As shown in Figure 9F, compared with the white bread without LW-CKFH, the LW-CKFH with bread had significantly lower glucose content during digestion. The glucose content in the LW-CKFH-bread after 2 h intestinal digestion was 170.75 mmol/L, while that of the bread without LW-CKFH was as high as 245.96 mmol/L. Obviously, within one hour of intestinal digestion, the glucose release rate of the bread with LW-CKFH was very slow, dropping by nearly 50% compared to the bread without LW-CKFH. This might be attributed to the binding of peptide fragments to digestive enzymes, thereby inhibiting α-glucosidase [20].

The kinetics of glucose release during the in vitro digestion of LW-CKFH bread reflect its potential ability to control the rate of sugar level released from food. Our results showed that LW-CKFH could inhibit the activity of α-glucosidase, thereby slowing down the hydrolysis rate of food starch. This was helpful for T2DM patients to control the sharp rise in post-meal blood glucose levels. In conclusion, our study demonstrated that LW-CKFH was a promising anti-diabetic component, with properties suitable for developing hypoglycemic foods or low glycemic index foods.

### 3.5. Identification and Screening of α-Glucosidase Inhibitory Peptides from LW-CKFH

Cell and animal studies had confirmed that LW-CKFH exhibited significant hypoglycemic effects. To analyze the structural characteristics of this fraction, LW-CKFH was isolated and purified by gel filtration chromatography, yielding four purified fractions (Figure 9A, F1–F4). Among these, fraction F2 exhibited the highest α-glucosidase inhibitory activity (Figure 9B). At a concentration of 5.0 mg/mL, F2 achieved 76.57% of the α-glucosidase inhibitory effect of acarbose (Figure 9C). The half-maximal inhibitory concentration (IC_50_) of F2 against α-glucosidase was 0.13 ± 0.01 mg/mL, indicating its high inhibitory activity. The F2 fraction was selected for structural identification via LC-MS/MS, leading to the identification of 1036 peptide sequences. Using the Peptide Ranker potential bioactivity scoring system, 162 peptide sequences with scores greater than 0.5 and good solubility were screened. Subsequently, the ToxinPred and AllerTOP programs were used to predict the toxicity and sensitization potential of these peptides, identifying 91 non-toxic and non-sensitizing peptides. Hydrophobic amino acids tend to bind to the active site of α-glucosidase [44]. Previous studies have shown that peptides with high α-glucosidase inhibitory activity often share certain structural features in their amino acid composition. For example, the C-terminus of such peptides typically contains residues such as Arg, Lys, or Ala, while the N-terminus consists of Ser, Thr, Tyr, Lys, or Arg [45]. The presence of aliphatic amino acids such as Leu, Ile, Val, and Pro within the peptide helps enhance its inhibitory effect on α-glucosidase [5,46]. Therefore, for these 91 peptides, we further utilized the PepDraw prediction tool in conjunction with the aforementioned structural characteristics to screen 18 peptides with a high proportion of hydrophobic amino acids and a bioactivity score greater than 0.8. Binding energy is a key parameter for evaluating protein–ligand interactions. Generally, the lower the binding energy, the more stable the binding between the peptide and the receptor protein [47]. Using acarbose as a control, the binding stability of the peptides with α-glucosidase was analyzed through molecular docking simulation. The binding energy of acarbose to α-glucosidase is −7.6 kcal/mol [44]. Finally, from the 18 peptides, six peptides with binding energies lower than −7.6 kcal/mol were selected (Table 1). Based on the proportion of hydrophobic amino acids, three peptides—LPFPRPAGPR, FDLPAR, and ANVFNPR—with a hydrophobic amino acid proportion exceeding 50% were chosen for in vitro synthesis.

### 3.6. Analysis of the Inhibitory Activity and Binding Action of Oligopeptides to α-Glucosidase

To evaluate the activity of the three selected peptides, in vitro synthesis was conducted. The results showed that the order of α-glucosidase inhibitory activity of the three peptides was FDLPAR > ANVFNPR > LPFPRPAGPR (Figure 9D). To further understand the mechanism by which these three peptides exert α-glucosidase inhibitory activity, their spatial structures, hydrogen bonds, and hydrophobic interactions were analyzed using PyMOL (Version 2.5) and LigPlus (Version 2.3.1) software. As shown in Figure 9E, FDLPAR formed hydrogen bonds with four amino acid residues—Tyr389, Gly399, Asp333, and Ala378—in the active center of α-glucosidase. In addition, it formed hydrophobic interactions with 17 surrounding amino acid residues, including Pro230, Leu227, Val334, Met302, Ala229, Val335, Val380, Asn301, Glu271, Phe166, Gly228, Phe297, Arg400, Phe397, Lys398, Gly402, and Thr339. Figure 9F showed that, in the α-glucosidase active center, LPFPRPAGPR formed hydrogen bonds with amino acid residues Leu227, Arg340, Thr339, and Glu377. Moreover, Asp333, Lys398, Pro230, Leu300, Val335, Glu396, Gly399, Met302, Phe297, Ala229, Arg429, and Tyr389 were part of the 18 adjacent amino acids involved in the hydrophobic interactions of LPFPRPAGPR. The peptide ANVFNPR formed hydrogen bonds with three amino acid residues (Lys225, Gly399, and Glu377) in α-glucosidase and also formed hydrophobic interactions with 16 surrounding amino acid residues (e.g., Asp401, Phe397, Ala229, and Val334) (Figure 9G). These results indicated that hydrogen bonds and hydrophobic interactions were the key forces driving the stable binding of the peptide to the active center of α-glucosidase. Interestingly, Gly399 and Glu377 were involved in the formation of almost all hydrogen bonds. Glycine combines with the carboxyl group of the glutamic acid side chain through the amide group and carbonyl group on its main chain, along with the highly electronegative oxygen atom in the carboxyl group. A similar situation was observed for Tyr389, Thr339, and Asp333, which might explain the ease of hydrogen bond formation. Based on these findings, it could be inferred that these amino acid residues likely represent key residues in the active center of α-glucosidase.

## 4. Conclusions

This study aimed to explore the hypoglycemic efficacy of α-glucosidase inhibitory peptides derived from coconut kernel fiber and to reveal the feasibility of application in functional foods. A preparation method to achieve CKFH with high α-glucosidase inhibitory activity was established through ultrasound-assisted dual-enzyme hydrolysis. The LW-CKFH fraction (1–3 kDa) exhibited the highest inhibitory activity against α-glucosidase, which could restore glucose metabolism in IR-HepG2 cells to 71.37% of normal levels. Moreover, LW-CKFH-H (800 mg/kg·bw) could improve insulin resistance by 77.2% relative to metformin. It was found that LW-CKFH effectively alleviated insulin resistance and enhanced insulin sensitivity (increasing it by 11.76% in T2DM mice) by repairing hepatocyte damage, promoting glucose metabolism, and further improving abnormal lipid metabolism and inflammatory responses. In addition, LW-CKFH had good resistance to gastrointestinal digestion, thermal stability, and acid-base tolerance. It could slow down the releasing rate of glucose in bread and reduced glucose production in bread by nearly 50% within the first hour of digestion. The LW-CKFH was more suitable as an ingredient for fruits, vegetables, grains, and dairy products to develop hypoglycemic foods. In addition, three oligopeptides (FDLPAR, LPFPRPAGPR, and ANVFNPR) with high α-glucosidase inhibitory activity were identified from LW-CKFH for the first time. Hydrogen bonds and hydrophobic interactions were found to be the main forces enabling the stable binding of these three oligopeptides to the α-glucosidase active site, which is probably the reason why the LW-CKFH had higher activity. In the future, it will be necessary to analyze the structure–activity relationships of these oligopeptides. This study provides a new strategy for the high-value utilization of CKF by-products.

## Figures and Tables

**Table 1:** Selected physicochemical properties of peptides in F2.

Sequence	Molwet (Da)	Bioactivity Peptide Ranker	Proportion of Hydrophobic Amino Acids (%)	Toxin	Allergenicity	Binding Energy (kcal/mol)
LPFPRPAGPR	1106.63	0.85	70	F	F	−7.9
FDLPAR	717.38	0.97	66.67	F	F	−7.8
ANVFNPR	816.42	0.93	57.14	F	F	−7.6
HFPERP	781.39	0.96	50	F	F	−7.7
FPAGSQRGAP	986.492	0.86	50	F	F	−7.9
RADVFNPRG	1031.25	0.7	44.44	F	F	−8

F indicates non-toxicity and sensitization.

## Data Availability

The original contributions presented in this study are included in the article. Further inquiries can be directed to the corresponding authors.

## References

[1] Zhou B., Rayner A., Gregg E.W., Sheffer K.E., Carrillo-Larco R.M., Bennett J.E., Shaw J.E., Paciorek C.J., Singleton R., Barradas-Pires A. (2024). Worldwide trends in diabetes prevalence and treatment from 1990 to 2022: A pooled analysis of 1108 population-representative studies with 141 million participants. Lancet.

[2] Shahwan M., Alhumaydhi F.A., Ashraf G.M., Hasan P.M.Z., Shamsi A. (2022). Role of polyphenols in combating type 2 diabetes and insulin resistance. Int. J. Biol. Macromol..

[3] Santos-Hernández M., Cermeño M., Recio I., FitzGerald R.J. (2021). In vitro dipeptidyl peptidase IV inhibitory activity and in situ insulinotropic activity of milk and egg white protein digests. Food Funct..

[4] Chen H., Li W., Hu W., Liu J., Zhang C., Wang Y., Zhang C., Zhang X., Chen S., Nie Q. (2025). Discovery of a novel tetrapeptide as glucose homeostasis modulator with bifunctionalities of targeting DPP-IV and microbiota. iMeta.

[5] Mu X., Wang R., Cheng C., Ma Y., Zhang Y., Lu W. (2023). Preparation, structural properties, and in vitro and in vivo activities of peptides against dipeptidyl peptidase IV (DPP-IV) and α-glucosidase: A general review. Crit. Rev. Food Sci. Nutr..

[6] Fadimu G.J., Farahnaky A., Gill H., Olalere O.A., Gan C.Y., Truong T. (2022). In-silico analysis and antidiabetic effect of α-Amylase and α-Glucosidase inhibitory peptides from Lupin protein hydrolysate: Enzyme-peptide interaction study using molecular docking approach. Foods.

[7] Li W., Xu T., Yuan Y., Tang S., Wu T., Pan S., Xu X. (2025). Extraction and activity mechanism of α-Glucosidase inhibitory peptides from Seabuckthorn seed meal. J. Food Sci..

[8] Zhang M., Chen C., Wei F., Zhao N., Yang W., Tianrong Z., Ren G., Zhijun Q., Bin Z. (2025). Identification and molecular mechanism of novel α-Glucosidase inhibitory peptides from the hydrolysate of hemp seed proteins: Peptidomic analysis, molecular docking, and dynamics simulation. Int. J. Mol. Sci..

[9] Li L., Sun J., Wanting Z., Yuanyuan Z., Wang C. (2025). Purification and molecular docking of α-glucosidase inhibitory peptides from mung bean protein hydrolysates. LWT.

[10] Hau E.H., Chew L.Y., Yeo S.K., Owatworakit A., Teh S.S., Mah S.H. (2024). Oil palm leaf protein hydrolysate and its novel peptides as alternative plant-based α-glucosidase inhibitors. Int. J. Biol. Macromol..

[11] Zhou S., Zhang Z., Li S., Liu M., Zhang Z., Wang N., Sun J. (2025). Characterization and efficacy of α-glucosidase inhibitory peptides from enzymatically hydrolyzed Peanut meal. Food Res. Int..

[12] Bian M.M., Yang Y., Liu Y., Xing J., Tu Y. (2025). Novel α-glucosidase inhibitory peptide derived from *Cyperus esculentus* meal protein hydrolysates: Screening, inhibition mechanism and stability. Food Bioprod. Process..

[13] Al-Khaza’leh J.F., Obeidat B.S. (2025). Effects of coconut meal inclusion on growth performance, nutrient utilization, carcass characteristics, and meat quality in Awassi Lambs. Vet. World.

[14] Wang L.N., Zhang F., Suo B., Han C., Ma Q., Sun J., Wang W. (2025). α-Glucosidase inhibitory peptides from the enzymolysis of Semen Ziziphi Spinosae protein using an ultrasound-assisted protease: Preparation and inhibitory mechanism. Food Res. Int..

[15] Kim J.S., Hyun T.K., Kim M.J. (2011). The inhibitory effects of ethanol extracts from sorghum, foxtailmillet and proso millet on α-glucosidase and α-amylase activities. Food Chem..

[16] Lu Y.C., Wang X., Wu Y., Wang Z., Zhou N., Li J., Shang X.Y., Lin P. (2022). Chemical characterization of the antioxidant and α-glucosidase inhibitory active fraction of *Malus* transitoria leaves. Food Chem..

[17] Nga N.T., Ngoc T.T.B., Nguyen T.T.M., Tran T.L., Thảo Đ.T.P. (2020). Optimization and application of MTT assay in determining density of suspension cells. Anal. Biochem..

[18] Qin J., Zheng X., Wang D., Song C., Shi W., Lu Y. (2025). Degradation polysaccharide of *Euryale ferox Salisb.* Seeds significantly enhanced the hypoglycemic efficiency of type 2 diabetes mellitus. Int. J. Biol. Macromol..

[19] Cai L., Wu S., Jia C., Cui C., Sun-Waterhouse D. (2023). Active peptides with hypoglycemic effect obtained from hemp (*Cannabis sativa* L) protein through identification, molecular docking, and virtual screening. Food Chem..

[20] Devi M.B., Chakraborty S., Nickhil C., Deka S.C. (2023). Effect of *Euryale ferox* seed shell extract addition on the in vitro starch digestibility and predicted glycemic index of wheat-based bread. Int. J. Biol. Macromol..

[21] Minekus M., Alminger M., Alvito P., Ballance S., Bohn T., Bourlieu-Lacanal C., Carrière F., Boutrou R., Corredig M., Dupont D. (2014). A standardised static in vitro digestion method suitable for food—An international consensus. Food Funct..

[22] Li Y., Zhang F., Gong J.S., Peng C. (2023). Two novel dipeptidyl peptidase-IV (DPP-IV) inhibitory peptides identified from truffle (*Tuber sinense*) by peptidomics, in silico, and molecular docking analysis. J. Food Compos. Anal..

[23] Trott O., Olson A.J. (2009). AutoDock Vina: Improving the speed and accuracy of docking with a new scoring function, efficient optimization, and multithreading. J. Comput. Chem..

[24] Jiang S., Li H., Zhang L., Mu W., Zhang S., Chen T., Wu J., Tang H., Zheng S., Liu Y. (2024). Generic Diagramming Platform (GDP): A comprehensive database of high-quality biomedical graphics. Nucleic Acids Res..

[25] Yang X., Wang D., Dai Y., Zhao L., Wang W., Ding X. (2023). Identification and molecular binding mechanism of novel α-glucosidase inhibitory peptides from Hot-pressed peanut meal protein hydrolysates. Foods.

[26] Wang L., He Y., Chen L., Ma X. (2022). Optimization of preparation of *Candida utilis* polypeptide by ultrasonic pretreatment and double enzyme method. Biomass Convers. Biorefin..

[27] Saleem R., Ahmad R. (2016). Effect of low frequency ultrasonication on biochemical and structural properties of chicken actomyosin. Food Chem..

[28] Ujiroghene O.J., Liu L., Zhang S., Lu J., Ujiroghene O.J., Lv J. (2019). α-Glucosidase and ACE dual inhibitory protein hydrolysates and peptide fractions of sprouted quinoa yoghurt beverages inoculated with *Lactobacillus* casei. Food Chem..

[29] Zhao Q., Wei G., Li K., Duan S., Ye R., Huang A. (2022). Identification and molecular docking of novel α-glucosidase inhibitory peptides from hydrolysates of Binglangjiang buffalo casein. LWT.

[30] Li Y., Fan Y., Liu J., Meng Z., Huang A., Xu F., Wang X. (2023). Identification, characterization and in vitro activity of hypoglycemic peptides in whey hydrolysates from rubing cheese by-product. Food Res. Int..

[31] Jiao Y., Wang X., Jiang X., Kong F., Wang S., Yan C. (2017). Antidiabetic effects of *Morus alba* fruit polysaccharides on high-fat diet and streptozotocin-induced type 2 diabetes in rats. J. Ethnopharmacol..

[32] Okita K., Iwahashi H., Kozawa J., Okauchi Y., Funahashi T., Imagawa A., Shimomura I. (2013). Homeostasis model assessment of insulin resistance for evaluating insulin sensitivity in patients with type 2 diabetes on insulin therapy. Endocr. J..

[33] Haq A.U., Khalid U., Ullah N., Tariq H., Akhtar J., Ullah I. (2023). A cross-sectional study on prevalence and pattern of dyslipidemia and its associated factors among patients with type 2 diabetes mellitus. Pak. J. Med. Health Sci..

[34] Zhang Y., Wang M., Li P., Lv G., Yao J., Zhao L. (2024). Hypoglycemic effect of polysaccharides from *Physalis alkekengi* L. in type 2 diabetes mellitus mice. Biology.

[35] Liŭ D., Zhang Y., Liu Y., Hou L.-q., Li S., Tian H., Zhao T. (2018). Berberine modulates gut microbiota and reduces insulin resistance via the TLR4 signaling pathway. Exp. Clin. Endocrinol. Diabetes.

[36] Thomas M.C. (2022). The clustering of cardiovascular, renal, adipo-metabolic eye and liver disease with type 2 diabetes. Metabolism.

[37] Bai Z., Huang X.J., Wu G., Zhou Y., Deng X., Yang J., Yin J., Nie S. (2023). Hepatic metabolism-related effects of polysaccharides from red kidney bean and small black soybean on type 2 diabetes. Food Chem..

[38] Ma M., Liu H., Yu J., He S., Li P., Ma C., Zhang H., Xu L., Ping F., Li W. (2020). Triglyceride is independently correlated with insulin resistance and islet beta cell function: A study in population with different glucose and lipid metabolism states. Lipids Health Dis..

[39] Li J., He J., He H., Wang X., Zhang S., He Y., Zhang J., Yuan C., Wang H., Xu D. (2024). Sweet triterpenoid glycoside from *Cyclocarya paliurus* ameliorates obesity-induced insulin resistance through inhibiting the TLR4/NF-κB/NLRP3 inflammatory pathway. Curr. Res. Food Sci..

[40] Cheng Y., Liu Y., Chen D., Zhou Y., Yu S., Lin H., Liao C.K., Lin H., Xu P., Huang M. (2021). Dual effects of quercetin on protein digestion and absorption in the digestive tract. Food Chem..

[41] Rivero-Pino F., Espejo-Carpio F.J., Guadix E.M. (2020). Evaluation of the bioactive potential of foods fortified with fish protein hydrolysates. Food Res. Int..

[42] Nawaz A., Irshad S., Khan I.A., Khalifa I., Walayat N., Aadil R.M., Kumar M., Wang M., Chen F., Cheng K.W. (2022). Protein oxidation in muscle-based products: Effects on physicochemical properties, quality concerns, and challenges to food industry. Food Res. Int..

[43] Fujie S., Horii N., Kajimoto H., Yamazaki H., Inoue K., Iemitsu K., Uchida M., Arimitsu T., Shinohara Y., Sanada K. (2024). Impact of resistance training and chicken intake on vascular and muscle health in elderly women. J. Cachexia Sarcopenia Muscle.

[44] Han L., Xie T., Wu Q., Hu Z., Luo Y., Luo F. (2023). Alpha-glucosidase inhibitory peptides: Sources, preparations, identifications, and action mechanisms. Nutrients.

[45] Ibrahim M.A., Bester M.J., Neitz A.W.H., Gaspar A.R.M. (2017). Structural properties of bioactive peptides with α-glucosidase inhibitory activity. Chem. Biol. Drug Des..

[46] Ren Y., Liang K.F., Jin Y., Zhang M., Chen Y., Wu H., Lai F. (2016). Identification and characterization of two novel α-glucosidase inhibitory oligopeptides from hemp (*Cannabis sativa* L.) seed protein. J. Funct. Foods.

[47] Capriotti A.L., Cavaliere C., Foglia P., Piovesana S., Samperi R., Chiozzi R.Z., Laganà A. (2014). Development of an analytical strategy for the identification of potential bioactive peptides generated by in vitro tryptic digestion of fish muscle proteins. Anal. Bioanal. Chem..

